# SPTBN1 attenuates rheumatoid arthritis synovial cell proliferation, invasion, migration and inflammatory response by binding to PIK3R2

**DOI:** 10.1002/iid3.724

**Published:** 2022-11-16

**Authors:** Li‐ping Dai, Xiao‐dong Xu, Ting‐ting Yang, Zhi‐hua Yin, Zhi‐zhong Ye, Ya‐zhi Wei

**Affiliations:** ^1^ Department of Rheumatology Futian District Rheumatology Hospital Shenzhen Guangdong China

**Keywords:** inflammation, invasion, migration, PIK3R2, proliferation, rheumatoid arthritis, SPTBN1

## Abstract

**Background:**

As an autoimmune systemic disorder, rheumatoid arthritis (RA) features chronic inflammation as well as synovial infiltration of immune cells. This study was designed with the purpose of discussing the hidden mechanism of SPTBN1 and exploring favorable molecular‐targeted therapies.

**Methods:**

With the application of RT‐qPCR and western blot, the expressions of SPTBN1 and PIK3R2 before or after transfection were estimated. Besides, Cell Counting Kit‐8, Edu, wound healing, transwell, enzyme‐linked immunosorbent assay, and TUNEL were adopted for the evaluation of the viability, proliferation, migration, invasion, inflammatory response, and apoptosis of fibroblast‐like synoviocyte (FLS). In addition, the interaction of SPTBN1 and PIK3R2 was testified by applying immunoprecipitation (IP) and western blot was utilized for the assessment of migration‐, apoptosis‐, and PI3K/AKT signal‐related proteins.

**Results:**

It was discovered that SPTBN1 declined in RA synovial cells and its overexpression repressed the proliferation, migration, invasion, and inflammation of RA‐FLSs but promoted apoptosis. IP confirmed that SPTBN1 could bind to PIK3R2 in FLSs. To further figure out the hidden mechanism of SPTBN1 in RA, a series of functional experiments were carried out and the results demonstrated that the reduced expressions of MMP2, MMP9, IL‐8, IL‐1β, IL‐6, and Bcl2 as well as increased levels of Bax and cleaved caspase3 in SPTBN1‐overexpressed RA‐FLSs were reversed by PIK3R2 depletion, revealing that SPTBN1 repressed the migration and inflammation and promoted the apoptosis of RA‐FLSs via binding to PIK3R2. Results obtained from western blot also revealed that PIK3R2 interference ascended the contents of p‐PI3K and p‐AKT in SPTBN1‐overexpressed RA‐FLSs, implying that SPTBN1 repressed PI3K/AKT signal in RA via PIK3R2.

**Discussion:**

SPTBN1 alleviated the proliferation, migration, invasion, and inflammation in RA via interacting with PIK3R2.

## Graphical abstract

Role of SPTBN1 in rheumatoid arthritis binding to PIK3R2.

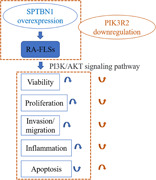

## 1 INTRODUCTION

Being a prevalent inflammatory disease, rheumatoid arthritis (RA) features progressive inflammatory injury of affected joints, thus contributing to cartilage destruction, bone erosion as well as disability.^1^ It was reported that 1.0% of the population suffers from RA in every corner of the world.^2^ The major clinical manifestations of RA include symmetrical polyarthritis with redness, swelling as well as pain in distal joints, particularly in the small joints of hands and feet.^3^ Previous studies have demonstrated that the risk factors contributing to RA include smoking, obesity, exposure to UV‐light, drugs, infection as well as sex hormones.^4^, ^5^, ^6^ Nevertheless, the specific cause of RA still remains obscure, which presents great challenges to the finding of favorable therapies for RA.^7^ In this way, the investigation of molecular‐targeted therapy for the improvement of RA is of great necessity.

SPTBN1, which is also named β2‐spectrin, belongs to the spectrin family and acts as an actin cross‐linked molecular skeleton protein, thus exserting its pivotal influence in arranging transmembrane proteins as well as organelle tissues.^8^ Accumulating studies have evidenced that SPTBN1 has involvement with the prognosis or advancement of human diseases. Take epithelial ovarian cancer (ECO) as an example, it was discovered that SPTBN1 was highly expressed in ECO cells and its upregulation repressed the migration as well as the growth of ECO cells.^9^ Additionally, SPTBN1 could promote the proliferation, differentiation and repress the apoptosis of osteoblasts, thus protecting against primary osteoporosis.^10^ Despite the fact that SPTBN1 has been widely discussed in many diseases, its role in RA still remains blank.

PIK3R2 protein acts as a member of the regulatory subunits of the class IA PI3K enzyme which can be triggered by tyrosine kinase receptors.^11^ A case of previous study held the opinion that the activity of PI3K could be inhibited when PIK3R2 bound to the catalytic subunit.^12^ More importantly, PIK3R2 expression was verified to be declined in RA.^13^ Besides, according to Biogrid database (https://thebiogrid.org/), SPTBN1 could interact with PIK3R2.

To sum up, the model of RA was established in vitro to study the role of SPTBN1 in RA as well as to discuss its hidden reaction mechanism, intending to find possible molecular‐targeted therapies for RA.

## 2 MATERIALS AND METHODS

### 2.1 Cell culture

NC‐fibroblast‐like synoviocytes (FLSs) and RA‐FLSs provided by Shanghai Yanyu Biotechnology Co., Ltd. were cultivated into DMEM (Guangdong Huan Kai Biotechnology Co., Ltd.) which was exposed to 10% fetal bovine serum (FBS; Guangzhou Perseco Biotechnology Co., Ltd.) as well as 1% antibiotics and was maintained at 37°C with 5% CO_2_.

### 2.2 Cell transfection

Plasmids carrying SPTBN1 (Ov‐SPTBN1), small interfering RNA (si‐RNA) targeting PIK3R2 (siRNA‐PIK3R2‐1 and siRNA‐PIK3R2‐2) as well as their corresponding negative control (Ov‐NC and siRNA‐NC) were supplied by GeneChem. The transfection of above plasmids into RA‐FLSs was implemented with the help of Lipofectamine 3000 (Wuhan Kehaojia Biotechnology Co., Ltd.). Subsequently, RT‐qPCR as well as western blot was adopted for the test of transfection efficacy.

### 2.3 Reverse transcription‐quantitative PCR

The RNA that was isolated from RA‐FLSs with TRIzol® reagent (Guangzhou Saiyan Biotechnology Co., Ltd.) was reversely transcribed into cDNA utilizing a commercial RevertAid™ cDNA Synthesis kit (Beijing Zhijie Fangyuan Technology Co., Ltd.). With the application of SYBR Green Master Mix (Applied Biosystems), quantitative real‐time PCR was implemented on the ABI PRISM 7900 Sequence Detection System (Applied Biosystems). Finally, the comparative Ct method was adopted for the estimation of relative gene expressions.

### 2.4 Western blot

Proteins that separated from RA‐FLSs with RIPA lysis buffer (Shanghai Absin Biotechnology Co., Ltd.) were quantified with the application of a bicinchoninic acid (BCA) protein assay kit (Shanghai Yisheng Biotechnology Co., Ltd.). Then, the proteins were exposed to 8% SDS‐PAGE, after which were transferred to PVDF membranes. The membranes that sealed by 5% nonfat milk or 5% bovine serum albumin (BSA) were subjected to primary antibodies against SPTBN1 (ab124888; 1:1,000; Abcam), MMP2 (ab92536; 1:1,000; Abcam), MMP9 (ab76003; 1:1,000; Abcam), Bcl2 (ab32124; 1:1,000; Abcam), Bax (ab32503; 1:1,000; Abcam), cleaved caspase3 (ab32042; 1:500; Abcam), PIK3R2 (ab180967; 1:2,000; Abcam), p‐PI3K (ab278545; 1:1,000; Abcam), p‐AKT (ab38449; 1:1,000; Abcam), PI3K (ab140307; 1:1,000; Abcam), AKT (ab8805; 1:1,000; Abcam), or GAPDH (ab9485; 1:2500; Abcam) at 4°C overnight, after which was the probe with HRP‐labeled goat anti‐rabbit secondary antibody (ab6759; 1:5000; Abcam) at room temperature for 2 h. At last, ECL (Yeasen Biotech) and ImageJ (Version 146) were applied for the visualization and analysis of protein blots.

### 2.5 Cell Counting Kit‐8 assay

RA‐FLSs were inoculated into 96‐well plates, following which was the cultivation for 24, 48, and 72 h. Subsequently, each well was added with a 10 μl Cell Counting Kit‐8 (CCK‐8) reagent (Beyotime) to further cultivate the cells. Finally, the OD value at 450 nm was decided with the aid of a microplate reader (Thermo Fisher Scientific).

### 2.6 5‐ethynyl‐2′‐deoxyuridine staining

RA‐FLSs that injected into six‐well plates were incubated overnight at room temperature. Afterward, 5‐ethynyl‐2′‐deoxyuridine (Edu) solution was put into wells for the further cultivation of RA‐FLSs. After the removal of working fluid, the digestion and centrifugation of cells were implemented. Then, the cells were subjected to 4% paraformaldehyde fixation as well as 0.5% Trionx‐100 permeation. Subsequently, RA‐FLSs were exposed to Click reaction solution for 30 min away from light. Finally, a fluorescence microscope was adopted for the capture of cell images.

### 2.7 Wound healing

After the inoculation into six‐well plates, RA‐FLSs were cultivated until cell fusion has achieved 90%–100%. With the aid of a pipette tip, a wound in the cell monolayer was created. Then, phosphate‐buffered saline (PBS)‐rinsed cells were incubated in an incubator which was placed at 37°C with 5% CO_2_. The record of cells was conducted at 0 and 24 h. Finally, the areas occupied by migrated cells were tracked employing ImageJ software.

### 2.8 Transwell

The invasive ability of RA‐FLSs was assessed with transwell invasion assay. Initially, RA‐FLSs were injected onto the upper chamber of the transwell which was decorated with Matrigel (BD Biosciences) while the medium exposed to 10% FBS was put on the lower chamber of the transwell. After 24 h, 4% paraformaldehyde as well as 0.1% crystal violet was applied for the fixation and staining of RA‐FLSs, respectively. Finally, the photographs of cells passing through the membranes were tracked utilizing a microscope.

### 2.9 Enzyme‐linked immunosorbent assay

With the aim of resolving the releases of inflammatory factors, the levels of interleukin‐8 (IL‐8), interleukin‐1 beta (IL‐1β), and interleukin‐6 (IL‐6) in cell supernatants were assessed. In the premise of λ = 450 nm, the OD value was estimated by employing a microplate reader (Bio‐Rad). The results were decided with the standard cure.

### 2.10 Terminal‐deoxynucleotidyl transferase‐mediated nick end labeling

The apoptosis of RA‐FLSs was evaluated by a terminal‐deoxynucleotidyl transferase‐mediated nick end labeling (TUNEL) assay kit (Invitrogen; Thermo Fisher Scientific) strictly in light of standard specifications. In brief, RA‐FLSs were initially subjected to 4% paraformaldehyde embedment and 0.25% Triton‐X 100 permeabilization. After the rinse with PBS three times, RA‐FLSs were exposed to TUNEL reaction solution for 1 h in line with the standard protocol. Thereafter, DAPI was applied for the staining of cell nuclei. At last, a florescent microscope was employed for the estimation of apoptotic cells in five randomly selected fields.

### 2.11 Immunoprecipitation

Proteins that separated with RIPA lysis buffer (Shanghai Absin Biotechnology Co., Ltd.) were quantified utilizing a BCA protein assay kit (Shanghai Yisheng Biotechnology Co., Ltd.). For immunoprecipitation (IP), proteins were exposed to specific antibodies against SPTBN1 and PIK3R2 overnight at 4°C. After that, Protein A/G PLUS‐Agarose beads (Invitrogen; Thermo Fisher Scientific, Inc.) were added to further cultivate the cells. Subsequently, the centrifugation of the collected beads was implemented at 12,000*g* for 2 min at 4°C. After the resuspension of precipitated proteins in 2× SDS‐PAGE loading buffer, the supernatant was rinsed from the beads. Finally, the IP of cells was visualized employing western blot.

### 2.12 Statistical analysis

All data displayed in the form of mean ± standard deviation (SD) got analyzed with GraphPad Prism 8.0 software (GraphPad Software, Inc.). One‐way analysis of variance followed by Tukey's post hoc test was utilized for the demonstration of comparisons among multiple groups while unpaired Student's *t*‐test was employed for the exhibition of differences between two groups. *p* < .05 meant that all experimental figures demonstrated statistically significant.

## 3 RESULTS

### 3.1 SPTBN1 was downregulated in RA‐FLSs

**Figure 1:**
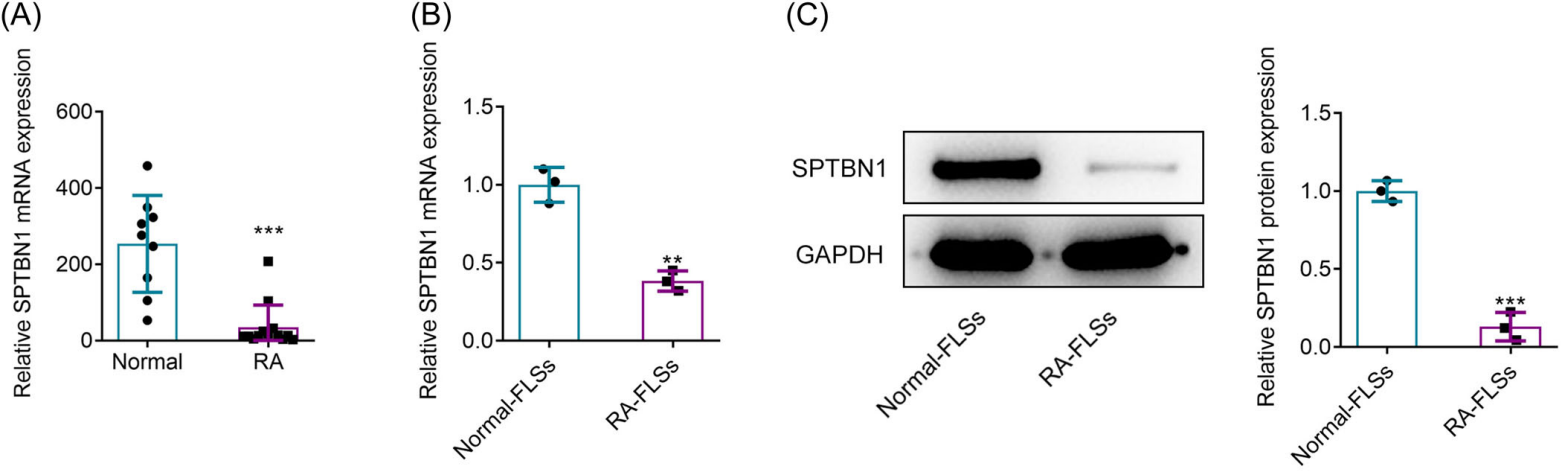
SPTBN1 was downregulated in RA‐FLSs. (A) GSE55457 database revealed the expression of SPTBN1 in RA patients. ***p < .001 versus Normal group. Normal group (*n* = 9) and RA group (*n* = 13). (B) The mRNA expression of SPTBN1 in Normal‐FLSs and RA‐FLSs was detected using RT‐qPCR. (C) The protein expression of SPTBN1 was detected in Normal‐FLSs and RA‐FLSs using western blot. **p < .01 and ***p < .001 versus Normal‐FLSs group. *n* = 3. student's t‐test. FLS, fibroblast‐like synoviocyte; RA, rheumatoid arthritis; RT‐qPCR, reverse transcription‐quantitative PCR.

According to GSE55457 database, SPTBN1 was reduced in patients suffering from RA (Figure 1A). In this study, reverse transcription‐quantitative PCR (RT‐qPCR) as well as western blot was applied for the assessment of SPTBN1 in RA‐FLSs. Results in Figure 1B,C demonstrated that the mRNA and protein expressions of SPTBN1 greatly declined in comparison with that in the Normal‐FLSs group. Evidently, this finding was consistent with the results in GSE55457 database.

### 3.2 SPTBN1 overexpression alleviated the proliferation, migration, and invasion of RA‐FLSs

**Figure 2:**
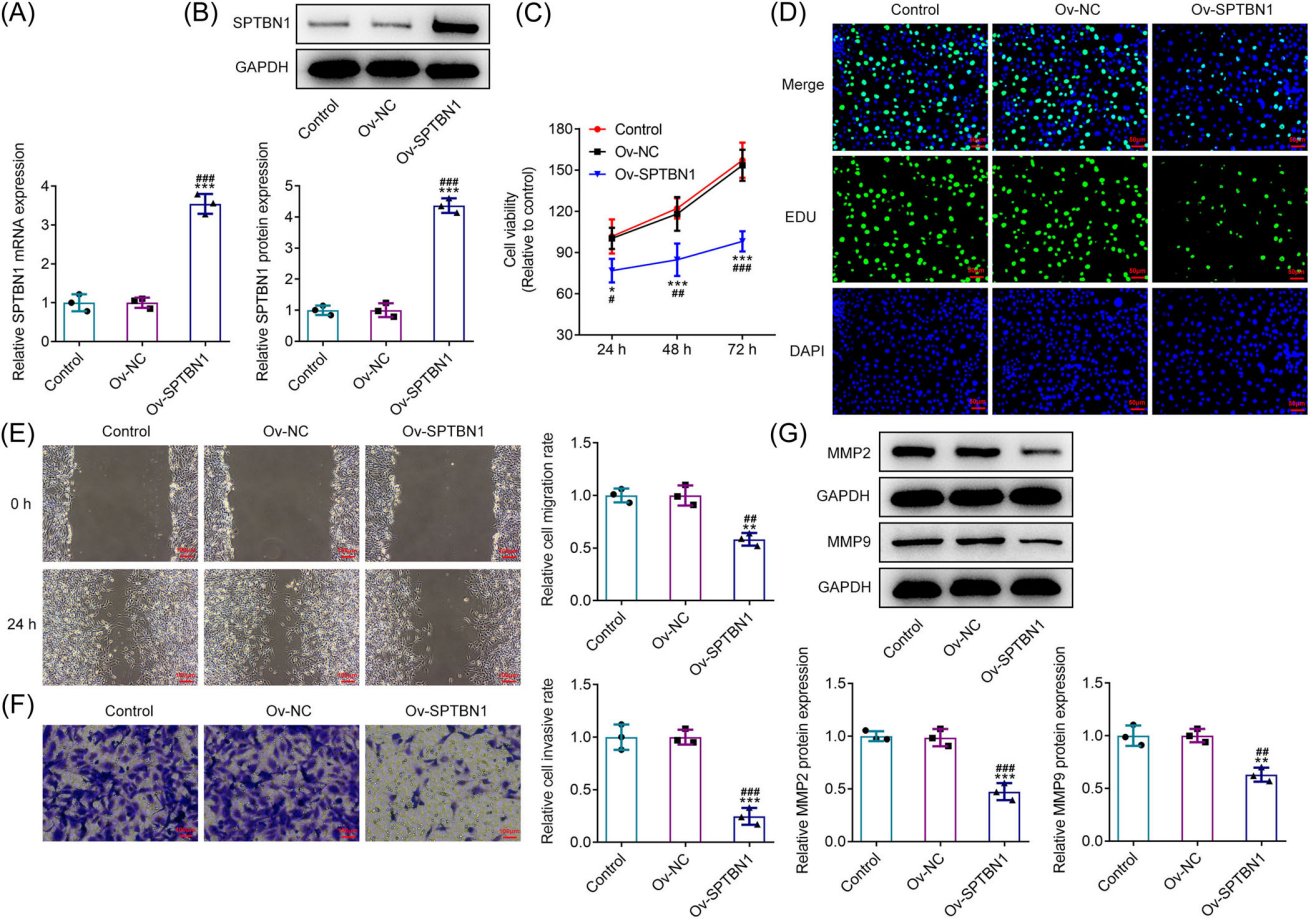
SPTBN1 overexpression alleviated the proliferation, migration, and invasion of RA‐FLSs. (A, B) The mRNA and protein expressions of SPTBN1 in RA‐FLSs transfected with Ov‐SPTBN1 were detected using RT‐qPCR and western blot. (C) The viability of RA‐FLSs transfected with Ov‐SPTBN1 was detected using CCK‐8. (D) The proliferation of RA‐FLSs transfected with Ov‐SPTBN1 was detected using Edu (magnification, 200×). (E, F) The migration and invasion of RA‐FLSs transfected with Ov‐SPTBN1 were detected using wound healing and transwell (magnification, 100×). (G) The expressions of MMP2 and MMP9 in RA‐FLSs transfected with Ov‐SPTBN1 were detected using western blot. *p < .05, **p < .01 and ***p < .001 versus Control group. ^#^ *p* < .05, ^##^ *p* < .01 and ^###^ *p* < .001 versus Ov‐NC group. *n* = 3. ANOVA followed by Tukey's test. ANOVA, analysis of variance; FLS, fibroblast‐like synoviocyte; RA, rheumatoid arthritis; RT‐qPCR, reverse transcription‐quantitative PCR.

To upregulate SPTBN1 expression, plasmids carrying SPTBN1 were transfected into RA‐FLSs and RT‐qPCR as well as western blot was adopted for the test of transfection efficacy. In contrast with the Ov‐NC group, the expressions of SPTBN1 at both mRNA and protein levels were markedly enhanced after overexpressing SPTBN1 expression (Figure 2A,B). To explore the impacts of SPTBN1 overexpression on cell viability and proliferation, CCK‐8 and Edu were employed. Results in Figure 2C,D revealed that SPTBN1 overexpression remarkably declined the cell viability and proliferation of RA‐FLSs when compared to the Ov‐NC. Additionally, the migration and invasion of SPTBN1‐overexpressed RA‐FLSs were estimated utilizing wound healing and transwell. As Figure 2E,F depicted, the migrative and invasive abilities of RA‐FLSs were conspicuously diminished after the transfection of the cells with SPTBN1 overexpression plasmids in comparison with that in the Ov‐NC group. Results obtained from western blot exhibited that the contents of MMP2 and MMP9 were dramatically cut down after overexpressing SPTBN1 when compared to the Ov‐NC group (Figure 2G). To sum up, the above findings indicated that SPTBN1 overexpression alleviated the proliferation, migration, and invasion of RA‐FLSs.

### 3.3 SPTBN1 overexpression alleviated the inflammation of RA‐FLSs but promoted the apoptosis

**Figure 3:**
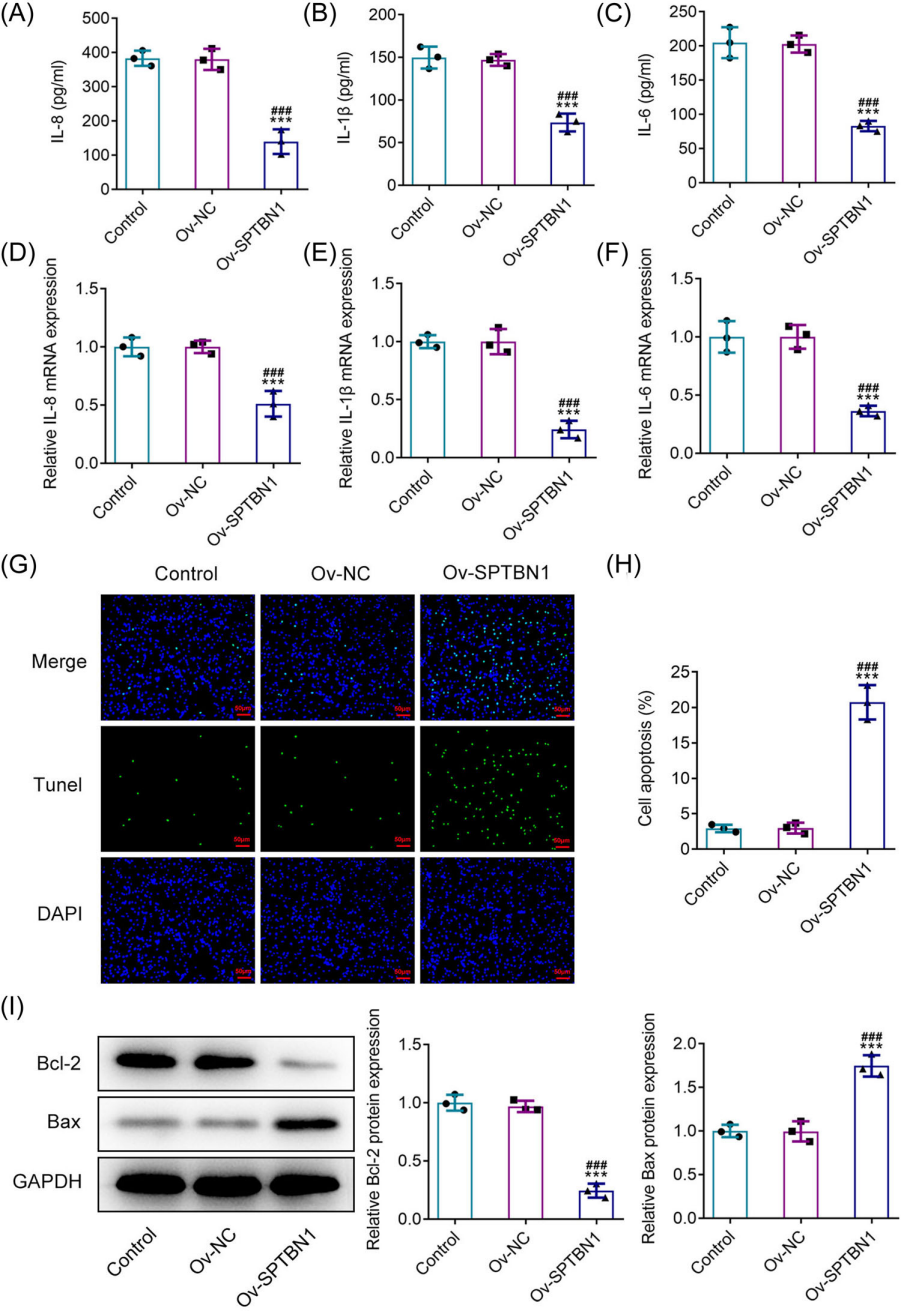
SPTBN1 overexpression alleviated the inflammation of RA‐FLSs but promoted apoptosis. (A–C) The levels of IL‐8, IL‐1β, and IL‐6 in RA‐FLSs transfected with Ov‐SPTBN1 were detected using ELISA. (D–F) The mRNA expressions of IL‐8, IL‐1β, and IL‐6 in RA‐FLSs transfected with Ov‐SPTBN1 were detected using RT‐qPCR. (H, G) The apoptosis of RA‐FLSs transfected with Ov‐SPTBN1 was detected using TUNEL (magnification, 200×). (I) The expressions of Bcl2, Bax, and cleaved caspase3 in RA‐FLSs transfected with Ov‐SPTBN1 were detected using western blot. ***p < .001 versus Control group. ^###^ *p* < .001 versus Ov‐NC group. *n* = 3. ANOVA followed by Tukey's test. ANOVA, analysis of variance; ELISA, enzyme‐linked immunosorbent assay; FLS, fibroblast‐like synoviocyte; RA, rheumatoid arthritis; RT‐qPCR, reverse transcription‐quantitative PCR; TUNEL, terminal‐deoxynucleotidyl transferase‐mediated nick end labeling.

With the aim of investigating the impacts of SPTBN1 overexpression on the inflammation of RA‐FLSs, the levels of IL‐8, IL‐1β, and IL‐6 were resolved. In comparison with the Ov‐NC group, SPTBN1 overexpression greatly descended the levels of IL‐8, IL‐1β, and IL‐6 (Figure 3A–C). Similarly, the mRNA levels of these inflammatory cytokines were also diminished by SPTBN1 overexpression, indicating that SPTBN1 overexpression exhibited suppressive impacts on the inflammation of RA‐FLSs (Figure 3D–F). In addition, TUNEL was employed to estimate the apoptosis of RA‐FLSs and the results showed that SPTBN1 overexpression tremendously ascended the apoptosis level of RA‐FLSs in contrast with the Ov‐NC group (Figure 3G,H). Furthermore, the upregulation of SPTBN1 reduced Bcl2 content but ascended the contents of Bax and cleaved caspase3 compared with the Ov‐NC group, suggesting the promoting impacts of SPTBN1 overexpression on RA‐FLSs apoptosis (Figure 3I).

### 3.4 SPTBN1 could bind to PIK3R2 in RA‐FLSs

**Figure 4:**
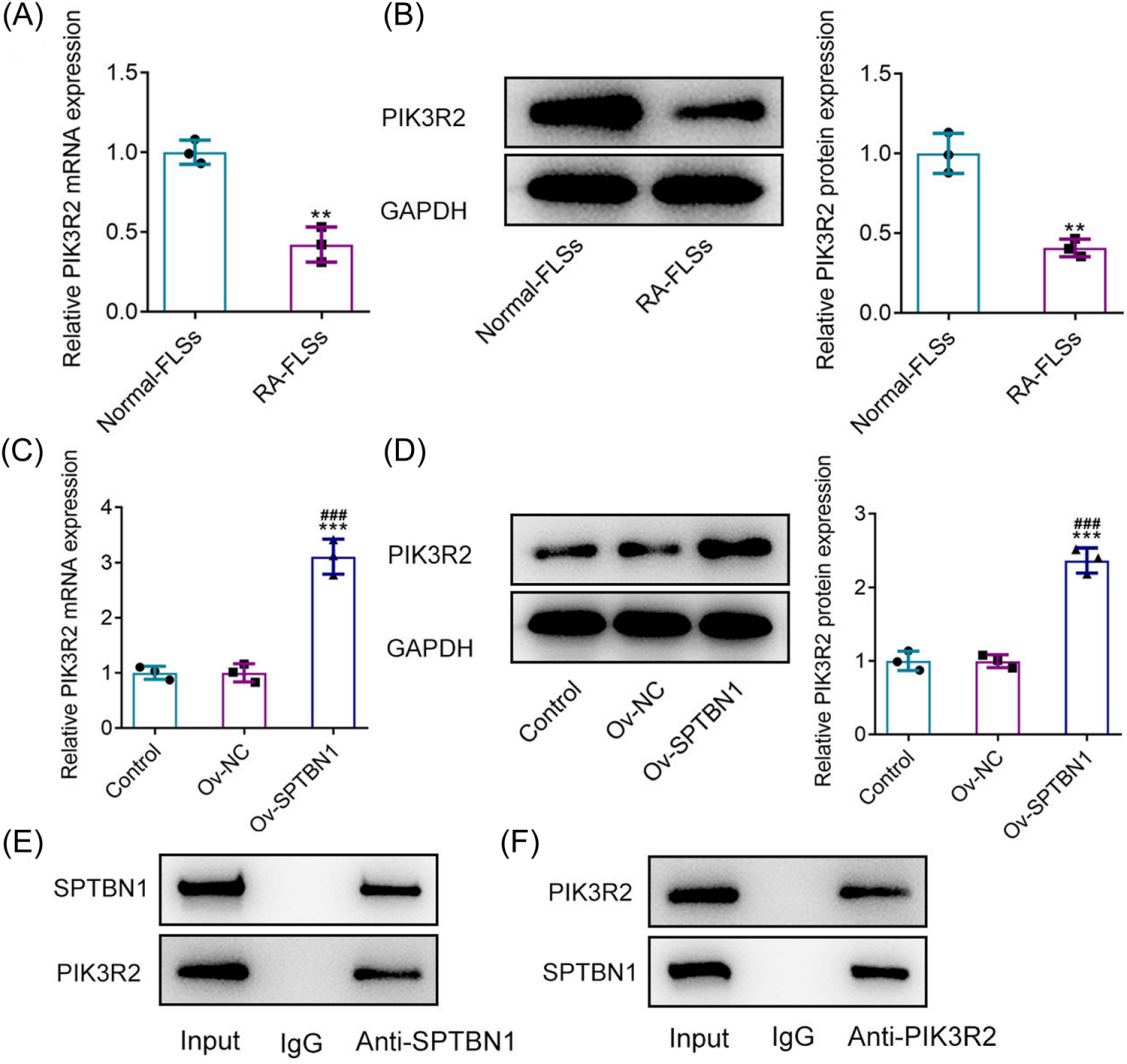
SPTBN1 could bind to PIK3R2 in RA‐FLSs. (A, B) The mRNA and protein expressions of PIK3R2 in Normal‐FLSs and RA‐FLSs were detected using RT‐qPCR and western blot. **p < .01 versus Normal‐FLSs group. *n* = 3. Student's *t*‐test. (C, D) The protein expression of PIK3R2 in RA‐FLSs transfected with Ov‐SPTBN1 was detected using RT‐qPCR and western blot. ***p < .001 versus Control group. ^###^ *p* < .001 versus Ov‐NC group. *n* = 3. ANOVA followed by Tukey's test. (E, F) The binding of SPTBN1 and PIK3R2 in RA‐FLSs was detected using IP. ANOVA, analysis of variance; FLS, fibroblast‐like synoviocyte; RA, rheumatoid arthritis; RT‐qPCR, reverse transcription‐quantitative PCR.

\In the beginning, RT‐qPCR as well as western blot was adopted to evaluate the mRNA and protein expressions of PIK3R2. Compared with the Normal‐FLSs group, PIK3R2 was significantly cut down in RA‐FLSs (Figure 4A,B). It was also testified that SPTBN1 overexpression conspicuously enhanced the content of PIK3R2 in comparison with the Ov‐NC group (Figure 4C,D). In addition, results in Figure 4E,F demonstrated that SPTBN1 had abundant enrichment in anti‐PIK3R2, showing that SPTBN1 could bind to PIK3R2 in RA‐FLSs.

### 3.5 SPTBN1 attenuated the proliferation, invasion, and migration of RA‐FLSs via PIK3R2

**Figure 5:**
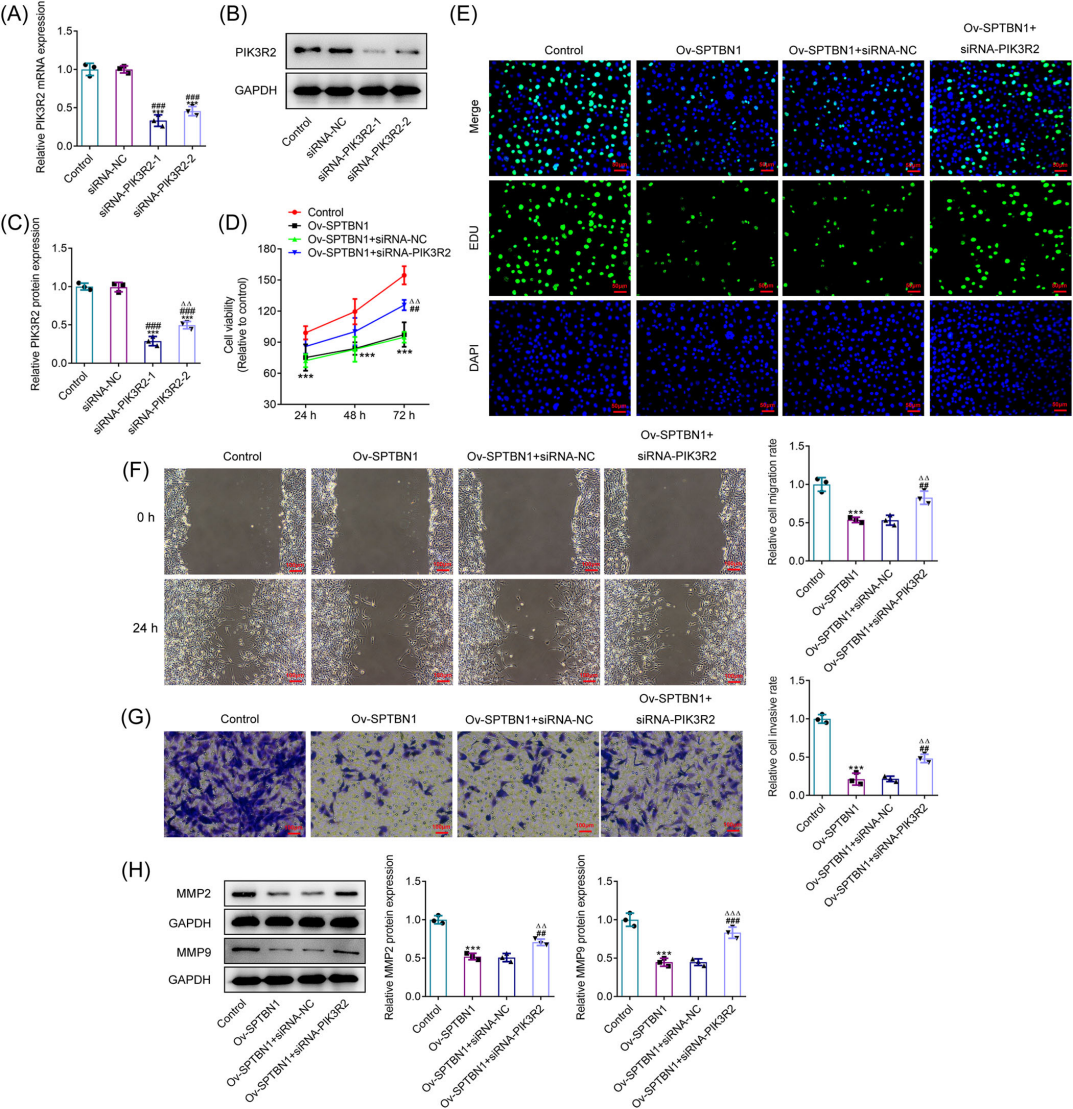
SPTBN1 attenuated the proliferation, invasion, and migration of RA‐FLSs via PIK3R2. (A–C) The expression of SPTBN1 in RA‐FLSs transfected with siRNA‐PIK3R2‐1 or 2 was detected using RT‐qPCR and western blot. ***p < .001 versus Control group. ^###^ *p* < .001 versus siRNA‐NC group. (D) The viability of RA‐FLSs transfected with Ov‐SPTBN1 and siRNA‐PIK3R2 was detected using CCK‐8. (E) The proliferation of RA‐FLSs transfected with Ov‐SPTBN1 and siRNA‐PIK3R2 was detected using Edu (magnification, 200×). (F, G) The migration and invasion of RA‐FLSs transfected with Ov‐SPTBN1 and siRNA‐PIK3R2 were detected using wound healing and transwell (magnification, 100×). (H) The expressions of MMP2 and MMP9 in RA‐FLSs transfected with Ov‐SPTBN1 and siRNA‐PIK3R2 were detected using western blot. ***p < .001 versus Control group. ^##^ *p* < .01 and ^###^ *p* < .001 versus Ov‐SPTBN1 group. ^∆∆^ *p* < .01 and ^∆∆∆^ *p* < .001 versus Ov‐SPTBN1 + siRNA‐NC group. *n* = 3. ANOVA followed by Tukey's test. ANOVA, analysis of variance; CCK‐8, Cell Counting Kit‐8; FLS, fibroblast‐like synoviocyte; RA, rheumatoid arthritis; RT‐qPCR, reverse transcription‐quantitative PCR.

To decline PIK3R2 expression, si‐RNA targeting PIK3R2 was adopted for the transfection of RA‐FLSs. In comparison with the siRNA‐NC group, the level of PIK3R2 was hugely diminished after the cell transfection with siRNA‐PIK3R2 (Figure 5A‐C). Notably, PIK3R2 had lower expression in RA‐FLSs transfected with siRNA‐PIK3R2‐1 than that in PIK3R2‐2‐silenced RA‐FLSs, in view of this, siRNA‐PIK3R2‐1 was adopted for ensuing studies. Compared with Ov‐SPTBN1 + siRNA‐NC, the declined cell viability and proliferation in RA‐FLSs caused by SPTBN1 overexpression were elevated by PIK3R2 interference (Figure 5D,E). Likewise, the reduced migrative and invasive capabilities of SPTBN1‐overexpressed RA‐FLSs were enhanced after silencing PIK3R2 expression (Figure 5F,G). Elsewhere, SPTBN1 overexpression greatly descended the contents of MMP2 and MMP9 when compared to the Control group, which were then ascended by PIK3R2 deficiency (Figure 5H). To conclude, the above results implied that SPTBN1 attenuated the proliferation, invasion, and migration of RA‐FLSs via PIK3R2.

### 3.6 SPTBN1 attenuated the inflammation and promoted the apoptosis of RA‐FLSs via PIK3R2

**Figure 6:**
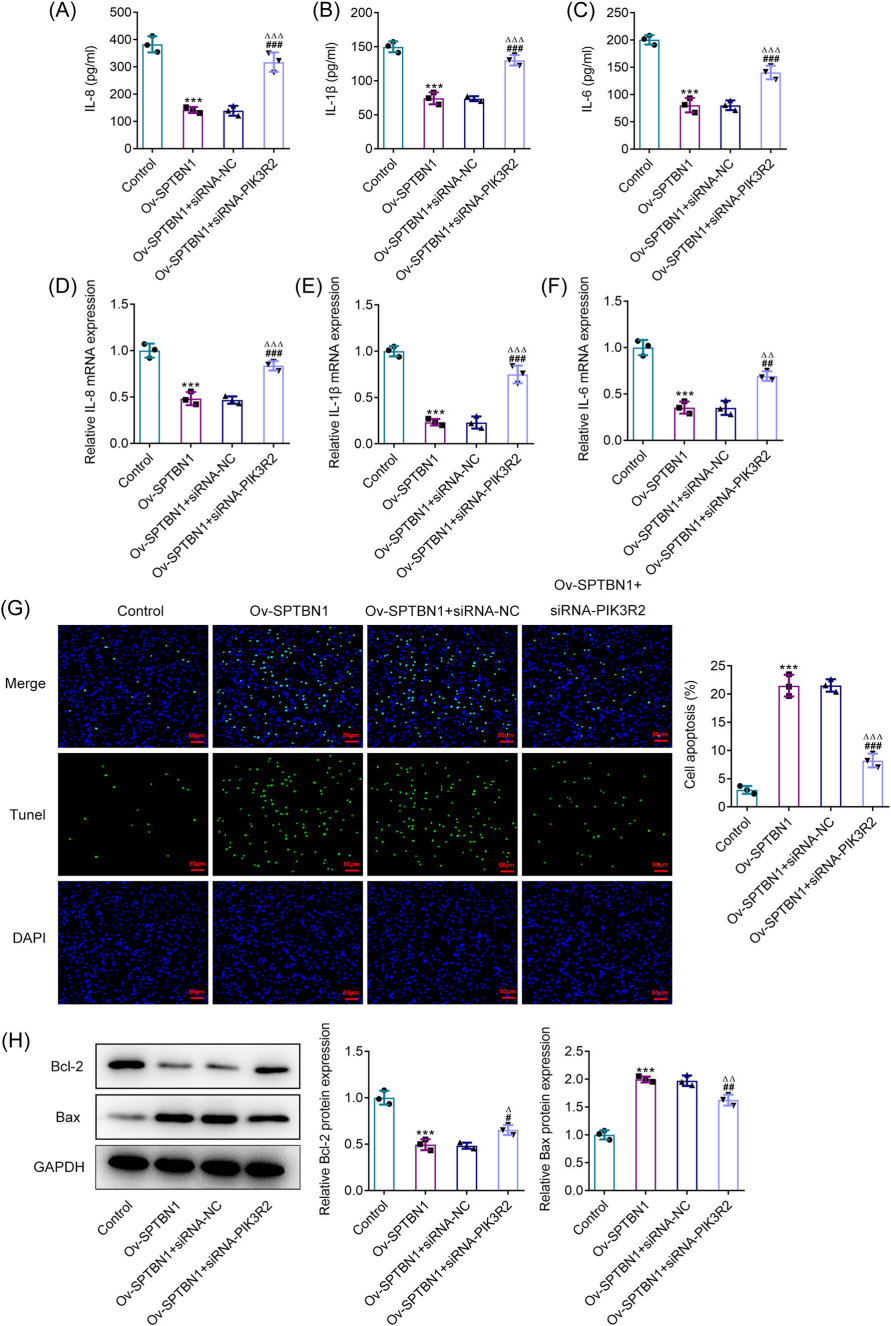
SPTBN1 attenuated the inflammation and promoted the apoptosis of RA‐FLSs via PIK3R2. (A–C) The levels of IL‐8, IL‐1β, and IL‐6 in RA‐FLSs transfected with Ov‐SPTBN1 and siRNA‐PIK3R2 were detected using ELISA. (D–F) The mRNA expressions of IL‐8, IL‐1β, and IL‐6 in RA‐FLSs transfected with Ov‐SPTBN1 and siRNA‐PIK3R2 were detected using RT‐qPCR. (G) The apoptosis of RA‐FLSs transfected with Ov‐SPTBN1 and siRNA‐PIK3R2 was detected using TUNEL (magnification, 200×). (H) The expressions of Bcl2, Bax, and cleaved caspase3 in RA‐FLSs transfected with Ov‐SPTBN1 and siRNA‐PIK3R2 were detected using western blot. ***p < .001 versus Control group. ^#^ *p* < .05, ^##^ *p* < .01 and ^###^ *p* < .001 versus Ov‐SPTBN1 group. ^∆^ *p* < .05, ^∆∆^ *p* < .01 and ^∆∆∆^ *p* < .001 versus Ov‐SPTBN1 + siRNA‐NC group. *n* = 3. ANOVA followed by Tukey's test. ANOVA, analysis of variance; ELISA, enzyme‐linked immunosorbent assay; FLS, fibroblast‐like synoviocyte; RA, rheumatoid arthritis; RT‐qPCR, reverse transcription‐quantitative PCR; TUNEL, terminal‐deoxynucleotidyl transferase‐mediated nick end labeling.

Results in Figure 6A–F exhibited that the levels of IL‐8, IL‐1β, and IL‐6 were greatly descended by SPTBN1 overexpression in contrast with that in the Control group, while PIK3R2 silenced imparted opposite impacts on these inflammatory cytokines, evidenced by the ascended levels of IL‐8, IL‐1β and IL‐6 in Ov‐SPTBN1 + siRNA‐PIK3R2 when compared to the Ov‐SPTBN1 + siRNA‐NC. Besides, the enhanced apoptosis level in SPTBN1‐overexpressed RA‐FLSs was reduced after the transfection of the cells with si‐RNA specific to PIK3R2 (Figure 6G). What is more, SPTBN1 overexpression cut down Bcl2 level but ascended the levels of Bax and cleaved caspase3, which were subsequently reversed by PIK3R2 interference (Figure 6H). In conclusion, SPTBN1 attenuated the inflammation and promoted the apoptosis of RA‐FLSs via PIK3R2.

### 3.7 SPTBN1 inhibited PI3K/AKT signaling expression in RA‐FLSs via PIK3R2

**Figure 7:**
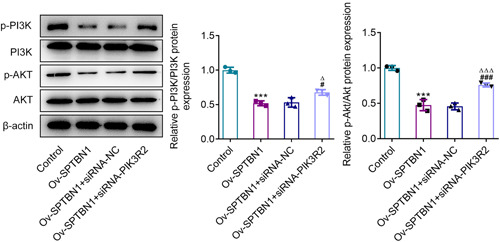
SPTBN1 inhibited PI3K/AKT signaling expression in RA‐FLSs via PIK3R2. The expressions of p‐PI3K, p‐AKT, PI3K, and AKT in RA‐FLSs transfected with Ov‐SPTBN1 and siRNA‐PIK3R2 were detected using western blot. ***p < .001 versus Control group. ^#^ *p* < .05 and ^###^ *p* < .001 versus Ov‐SPTBN1 group. ^∆^ *p* < .05 and ^∆∆∆^ *p* < .001 versus Ov‐SPTBN1 + siRNA‐NC group. *n* = 3. ANOVA followed by Tukey's test. ANOVA, analysis of variance; FLS, fibroblast‐like synoviocyte; RA, rheumatoid arthritis.

In comparison with Control group, SPTBN1 overexpression greatly cut down the contents of p‐PI3K and p‐AKT, which were subsequently enhanced by PIK3R2 depletion (Figure 7). Notably, SPTBN1 overexpression or PIK3R2 silence had no obvious impact on the expressions of PI3K and AKT. Collectively, the abovementioned results revealed that SPTBN1 inhibited PI3K/AKT signaling expression in RA‐FLSs via PIK3R2.

## 4 DISCUSSION

To the best of our knowledge, this study was the first to address the role of SPTBN1 in RA as well as its relationship with PIK3R2. In this paper, it was uncovered that SPTBN1 was reduced in RA‐FLSs, which was then elevated after the transfection of the cells with plasmids carrying SPTBN1. After overexpressing SPTBN1 expression, a series of cellular functional experiments were implemented and the results demonstrated that SPTBN1 overexpression repressed the proliferation, invasion, migration, and inflammation of RA‐FLSs but promoted apoptosis. Additionally, PIK3R2 was testified to be declined in RA‐FLSs and could interact with SPTBN1. After that, further experiments were carried out and the results revealed that SPTBN1 alleviated the proliferation, invasion, migration, inflammation and promoted the apoptosis of RA‐FLSs by binding to PIK3R2.

Apart from systems and organs, RA, which is a chronic inflammatory autoimmune disease and has varying severity among patients, is also involved in bones and joints.^14^ Being two fundamental properties of RA‐FLSs, proliferation as well as migration resulted in the pathology of RA.^15^ Besides, the focus on apoptosis of FLSs in RA was supposed to be an effective way to relieve RA.^16^ What is more, accumulating research have evidenced that inflammatory factors acted as a regulatory player in the proliferation, migration as well as apoptosis of RA‐FLSs.^17^ Considering this, the inhibition of inflammation, proliferation, and migration as well as the promotion of apoptosis might be a possible method to improve RA. As a critical cytoskeletal protein, SPTBN1 has involvement with many cellular processes.^18^ It was verified that SPTBN1 could be a novel therapeutic target for osteoporosis because SPTBN1 could facilitate the proliferative and differentiative capabilities of osteoblasts.^10^ Additionally, the depletion of SPTBN1 exhibited promotive effects on the migrative ability of triple‐negative breast cancer (TNBC), implying that SPTBN1 was a critical regulator in TNBC advancement.^19^ Moreover, the loss of SPTBN1 could contribute to the changes in cell apoptosis.^20^ In this paper, it was discovered that the mRNA and protein levels of SPTBN1 were greatly descended in RA‐FLSs. After overexpressing SPTBN1, the viability and proliferative ability of RA‐FLSs were suppressed. Elsewhere, SPTBN1 overexpression reduced the contents of MMP2, MMP9, IL‐8, IL‐1β, IL‐6, and Bcl2 but ascended the levels of Bax and cleaved caspase3 in RA‐FLSs, implying that SPTBN1 upregulation helped to repress the migration, inflammation and promoted the apoptosis of RA.

PIK3R2 is a subunit that regulates PI3K and Class I PI3K is composed of p110 catalytic subunit and p85 regulatory subunit.^21^ An increasing number of research have testified that PIK3R2 participates in various developmental processes through gene regulation. For instance, miR‐126 exhibited suppressive impacts on the proliferative, migrative, and invasive capabilities of non‐small‐cell lung cancer cells through targeting PIK3R2. Besides, Chen et al held the opinion that PTPN1suppressed the proliferation as well as metastasis in lung adenocarcinoma via mediating PIK3R2.^22^ Moreover, miR‐1226‐3p was verified to exsert protective impacts on breast cancer through targeting PIK3R2.^23^ Through our investigation, PIK3R2 was predicted to interact with SPTBN1 by Biogrid database. The strong affinity of SPTBN1 and PIK3R2 was testified by IP in this study and the results showed that SPTBN1 was enriched in anti‐PIK3R2, indicating that SPTBN1 could bind to PIK3R2 in RA‐FLSs. Additionally, the mRNA and protein levels of PIK3R2 were discovered to be descended in RA‐FLSs. To further discuss the mechanism of SPTBN1 in RA, functional experiments were carried out and it was found that SPTBN1 alleviated the proliferation, migration, invasion, inflammation and promoted the apoptosis of RA‐FLSs via targeting PIK3R2, thus protecting against RA. What is more, SPTBN1 was also evidenced to suppress PI3K/AKT signal in RA‐FLSs by binding PIK3R2.

However, this study also has limitations. Only cell model was used in this experiment and animal model would be conducted in future. Except for PI3K/AKT signal, other signaling pathways could be explored whether they were regulated by SPTBN1.

## 5 CONCLUSION

To sum up, this paper uncovered the regulatory impacts of SPTBN1 on the inflammation, proliferation, migration, invasion, and apoptosis of RA and identified that SPTBN1 could bind to PIK3R2, which for the first time revealed the mechanism by which SPTBN1 alleviates the advancement of RA.

## AUTHOR CONTRIBUTIONS

Ya‐zhi Wei designed and conceived the study. Li‐ping Dai, Xiao‐dong Xu, and Ting‐ting Yang conducted the experiments and all authors analyzed the data. Li‐ping Dai drafted the manuscript which was polished by Zhi‐hua Yin and Zhi‐zhong Ye. All authors have confirmed the authenticity of all the raw data and read and approved the final manuscript.

## CONFLICT OF INTEREST

The authors declare no conflict of interest.

## Data Availability

The data sets used and/or analyzed during the current study are available from the corresponding author upon reasonable request.

## References

[1] Smolen JS , Aletaha D , McInnes IB . Rheumatoid arthritis. Lancet. 2016;388(10055):2023‐2038. 27156434 10.1016/S0140-6736(16)30173-8

[2] Lawrence RC , Felson DT , Helmick CG , et al. Estimates of the prevalence of arthritis and other rheumatic conditions in the United States. Part II. Arthritis Rheum. 2008;58(1):26‐35. 18163497 10.1002/art.23176 PMC3266664

[3] Davis JM 3rd , Matteson EL , American College of Rheumatology; European League Against Rheumatism . My treatment approach to rheumatoid arthritis. Mayo Clin Proc. 2012;87(7):659‐673. 22766086 10.1016/j.mayocp.2012.03.011 PMC3538478

[4] Littlejohn EA , Monrad SU . Early diagnosis and treatment of rheumatoid arthritis. Prim Care. 2018;45(2):237‐255. 29759122 10.1016/j.pop.2018.02.010

[5] Myasoedova E , Crowson CS , Kremers HM , Therneau TM , Gabriel SE . Is the incidence of rheumatoid arthritis rising?: results from Olmsted County, Minnesota, 1955‐2007. Arthritis Rheum. 2010;62(6):1576‐1582. 20191579 10.1002/art.27425 PMC2929692

[6] Tan EM , Smolen JS . Historical observations contributing insights on etiopathogenesis of rheumatoid arthritis and role of rheumatoid factor. J Exp Med. 2016;213(10):1937‐1950. 27621417 10.1084/jem.20160792 PMC5030811

[7] Hyndman IJ . Rheumatoid arthritis: past, present and future approaches to treating the disease. Int J Rheum Dis. 2017;20(4):417‐419. 26845360 10.1111/1756-185X.12823

[8] Susuki K , Zollinger DR , Chang KJ , et al. Glial betaII spectrin contributes to paranode formation and maintenance. J Neurosci. 2018;38(27):6063‐6075. 29853631 10.1523/JNEUROSCI.3647-17.2018 PMC6031582

[9] Chen M , Zeng J , Chen S , et al. SPTBN1 suppresses the progression of epithelial ovarian cancer via SOCS3‐mediated blockade of the JAK/STAT3 signaling pathway. Aging. 2020;12(11):10896‐10911. 32516133 10.18632/aging.103303 PMC7346039

[10] Xu X , Yang J , Ye Y , et al. SPTBN1 prevents primary osteoporosis by modulating osteoblasts proliferation and differentiation and blood vessels formation in bone. Front Cell Dev Biol. 2021;9:653724. 33816505 10.3389/fcell.2021.653724 PMC8017174

[11] Liu LY , Wang W , Zhao LY , et al. Mir‐126 inhibits growth of SGC‐7901 cells by synergistically targeting the oncogenes PI3KR2 and Crk, and the tumor suppressor PLK2. Int J Oncol. 2014;45(3):1257‐1265. 24969300 10.3892/ijo.2014.2516

[12] Haapalainen AM , Daddali R , Hallman M , Ramet M . Human CPPED1 belongs to calcineurin‐like metallophosphoesterase superfamily and dephosphorylates PI3K‐AKT pathway component PAK4. J Cell Mol Med. 2021;25(13):6304‐6317. 10.1111/jcmm.16607 PMC8366450 34009729

[13] Gao J , Zhou XL , Kong RN , Ji LM , He LL , Zhao DB . microRNA‐126 targeting PIK3R2 promotes rheumatoid arthritis synovial fibro‐blasts proliferation and resistance to apoptosis by regulating PI3K/AKT pathway. Exp Mol Pathol. 2016;100(1):192‐198. 26723864 10.1016/j.yexmp.2015.12.015

[14] McInnes IB , Schett G . The pathogenesis of rheumatoid arthritis. N Engl J Med. 2011;365(23):2205‐2219. 22150039 10.1056/NEJMra1004965

[15] Liu F , Feng XX , Zhu SL , et al. Sonic hedgehog signaling pathway mediates proliferation and migration of fibroblast‐like synoviocytes in rheumatoid arthritis via MAPK/ERK signaling pathway. Front Immunol. 2018;9:2847. 30568656 10.3389/fimmu.2018.02847 PMC6290332

[16] Yang X , Chang Y , Wei W . Emerging role of targeting macrophages in rheumatoid arthritis: focus on polarization, metabolism and apoptosis. Cell Prolif. 2020;53(7):e12854. 32530555 10.1111/cpr.12854 PMC7377929

[17] Yang B , Ge Y , Zhou Y , et al. miR‐124a inhibits the proliferation and inflammation in rheumatoid arthritis fibroblast‐like synoviocytes via targeting PIK3/NF‐kappaB pathway. Cell Biochem Funct. 2019;37(4):208‐215. 30941802 10.1002/cbf.3386

[18] Chen S , Wu H , Wang Z , et al. Loss of SPTBN1 suppresses autophagy via SETD7‐mediated YAP methylation in hepatocellular carcinoma initiation and development. Cell Mol Gastroenterol Hepatol. 2022;13(3):949‐973.e7. 34737104 10.1016/j.jcmgh.2021.10.012 PMC8864474

[19] Wu H , Chen S , Liu C , et al. SPTBN1 inhibits growth and epithelial‐mesenchymal transition in breast cancer by downregulating miR‐21. Eur J Pharmacol. 2021;909:174401. 34358482 10.1016/j.ejphar.2021.174401

[20] Yang P , Yang Y , Sun P , et al. betaII spectrin (SPTBN1): biological function and clinical potential in cancer and other diseases. Int J Biol Sci. 2021;17(1):32‐49. 33390831 10.7150/ijbs.52375 PMC7757025

[21] Rathinaswamy MK , Burke JE . Class I phosphoinositide 3‐kinase (PI3K) regulatory subunits and their roles in signaling and disease. Adv Biol Regul. 2020;75:100657. 31611073 10.1016/j.jbior.2019.100657

[22] Chen Y , Tang J , Lu T , Liu F . CAPN1 promotes malignant behavior and erlotinib resistance mediated by phosphorylation of c‐Met and PIK3R2 via degrading PTPN1 in lung adenocarcinoma. Thorac Cancer. 2020;11(7):1848‐1860. 32395869 10.1111/1759-7714.13465 PMC7327690

[23] Mohamadzade Z , Soltani BM , Ghaemi Z , Hoseinpour P. Cell specific tumor suppressor effect of Hsa‐miR‐1226‐3p through downregulation of HER2, PIK3R2, and AKT1 genes. Int J Biochem Cell Biol. 2021;134:105965. 33675995 10.1016/j.biocel.2021.105965

